# Detection of Secondary Causes and Coexisting Diseases in Hypertensive Patients: OSA and PA Are the Common Causes Associated with Hypertension

**DOI:** 10.1155/2017/8295010

**Published:** 2017-06-13

**Authors:** Lei Wang, Nanfang Li, Xiaoguang Yao, Guijuan Chang, Delian Zhang, Mulalibieke Heizhati, Menghui Wang, Qin Luo, Jianqiong Kong

**Affiliations:** Hypertension Institute of Xinjiang, Hypertension Center of the People's Hospital of Xinjiang Uygur Autonomous Region, Urumqi, China

## Abstract

**Background:**

Since the control rate of blood pressure is lower in mainland China, the aim of this study is to investigate the proportion of secondary causes and coexisting diseases of hypertension in hypertensive patients.

**Methods:**

Data on consecutive patients with hypertension who visited the Hypertension Center. Diseases were detected using an established strict screening protocol.

**Results:**

Detection rate of secondary causes and coexisting diseases of hypertension was 39.5% among 3003 hypertensive patients. Obstructive sleep apnea (OSA) was the most common, accounting for 24.7% of patients, followed by primary aldosteronism (PA) (5.8%) and PA + OSA (4.9%). Endocrine hypertension accounted for 12.1% of patients, including 10.7% of patients with PA, 1.1% with hypothyroidism, 0.1% with pheochromocytoma, 0.1% with Cushing's syndrome, and 0.1% with hyperthyroidism, respectively. Those who smoke, those who are obese, and those who have diabetes accounted for 31.3%, 27.5%, and 16.6% of total patients, respectively. There were overlapping conditions in secondary causes and coexisting diseases of hypertension. OSA was the most common in each age- and BMI-stratified group.

**Conclusion:**

Findings from the current study suggest an increasing frequency of secondary forms of hypertension, highlighting the burden of OSA and PA in hypertensive patients.

## 1. Introduction

Hypertension is a major modifiable risk factor for morbidity and mortality of cardiocerebrovascular diseases and renal failure [1]. The control of blood pressure (BP) can reduce the risk of cardiocerebrovascular diseases, as confirmed by numerous studies and mentioned by a number of guidelines. However, the control rate of BP is lower although a lot of effort has been made [2]. A recent large-scale systemic analysis using data generated in mainland China between 2003 and 2012 showed that, among hypertensive patients, only 44.6% were aware of their condition, 35.2% were taking antihypertensive medication, and 11.2% achieved adequate BP control [3]. All this information poses the question why it is difficult for BP to reach target range and whether there are secondary causes and coexistence of some other diseases which evaluate BP and affect negatively BP control in hypertensive patients. Studies have shown that, in addition to genetic and environmental factors, a number of diseases increase BP, and their wide distributions in such various systems as endocrine and respiratory, renal, and vascular ones and psychological factors like anxiety have led to lower awareness or underestimation of these conditions by the cardiologists who mostly deal with hypertensive patients. A few studies on this aspect have mainly focused on the prevalence of secondary causes and coexisting diseases in resistant hypertension [4, 5]. Most importantly, the data are lacking, generated by comprehensive systemic specialty settings on secondary causes and coexisting diseases in hypertensive patients extensive in sample and investigated systematically.

The specialty center, Hypertension Center of a provincial general hospital of Xinjiang, Northwest of China, in which this study is conducted provides diagnosis and individualized treatment to hypertensive population in this region, particularly screening, diagnosis, and treatment service of secondary causes and coexisting diseases of hypertension including endocrine diseases such as primary aldosteronism, Cushing's syndrome, pheochromocytoma, and thyroid dysfunctions; respiratory diseases like obstructive sleep apnea; psychological factors such as anxiety; renal disease; and vascular diseases.

The aim of this study is to evaluate the proportion of secondary causes and coexisting diseases of hypertension in those who visited the center with the main complaint of elevated BP.

## 2. Materials and Methods

### 2.1. Patient Selection

Consecutive patients with hypertension referred to Hypertension Center of the People's Hospital of Xinjiang Uygur Autonomous Region, China, since January to December 2010, meeting the following inclusion criteria, were included into the current study. Inclusion criteria were as follows: age ≥ 18 years, new onset hypertension, previously diagnosed hypertension on antihypertensive treatment, or BP could not reach target (SBP < 140 mmHg and/or DBP < 90 mmHg) if without antihypertensive treatment. This study was approved by the Hospital Ethics Committee, and all patients provided written informed consent.

### 2.2. Office BP Measurements

Hypertension was defined according to the criteria established by the Seventh Report of the Joint National Committee on Prevention, Detection, Evaluation, and Treatment of High Blood pressure [6]. Office blood pressure measurement was based on the recommendation [7].

### 2.3. Primary Evaluation

**Figure 1:**
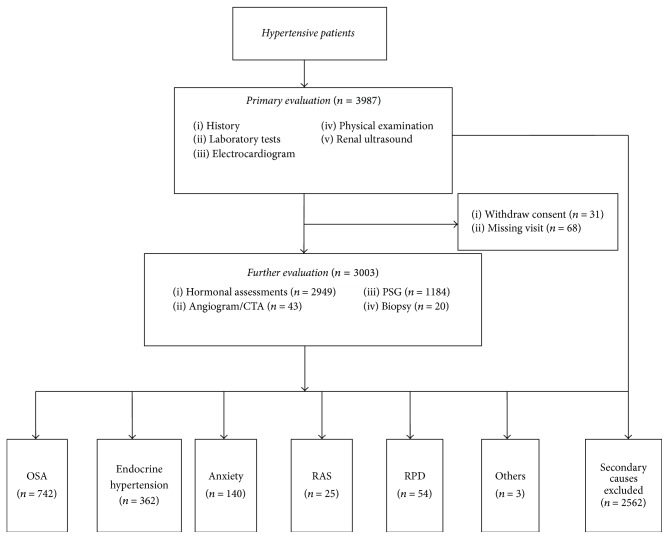
Study flow chart. PSG, polysomnography; CTA, computed tomography angiography; OSA, obstructive sleep apnea; RPD, renal parenchymal disease; RAS, renal artery stenosis.

A complete history and physical examination were performed by specialists in the clinic. Laboratory tests include measurements of serum electrolytes, creatinine, blood glucose, cholesterol (TC), triglycerides (TG), high density lipoprotein cholesterol (HDL-c), low density lipoprotein cholesterol (LDL-c), and hemoglobin, as well as tests for proteinuria and urinary sediment. Electrocardiogram and renal ultrasound were recorded in all patients. Patients with suspicious symptoms, signs, and laboratory examinations were suggested for further evaluation of secondary causes of hypertension, with particular attention given to the following: the age < 30 years at onset of hypertension, resistant hypertension, severe hypertension or hypertensive emergencies, sudden increase of BP in a previously stable patient, accelerated hypertension, severe target organ damage not matched for the duration of high BP, hypertension with adrenal incidentaloma, exaggerated diurnal sleepiness, nocturnal snoring, paroxysmal hypertension, hematuria, proteinuria, increased serum creatinine, hypokalemia, an abdominal bruit, particularly when diastolic, differences of >1.5 cm in the length between the two kidneys via renal ultrasound, and so forth [8]. The procedure for screening of secondary causes and coexisting diseases of hypertension was presented in Figure 1.

### 2.4. Further Evaluation for Secondary Causes and Coexisting Diseases of Hypertension

#### 2.4.1. Renal Parenchymal Disease (RPD)

Patients were diagnosed with hypertension after a renal disease history. These patients also had abnormalities of urinary sediment including hematuria and proteinuria, and serum creatinine in the upright position (>177 umol/L) and/or pathological diagnosis after kidney biopsy and/or imaging showed abnormalities in morphology and structure of kidney.

#### 2.4.2. Renal Artery Stenosis (RAS)

Renal artery stenosis was confirmed by angiogram and/or computed tomography angiography (CTA). Angiostenosis > 50% was confirmed positive [9].

#### 2.4.3. Primary Aldosteronism (PA)

The screening process and diagnosis of PA was made based on 2008 endocrine society's guideline [10] and on the previous studies conducted in our center [11]. A suppressed PRA (<1.0 ug·L^−1^·h^−1^) in addition to an elevated aldosterone level (>12 ng·L^−1^) or an ARR (the PAC to PRA ratio) greater than 20 ng/dl per ng/ml/h was considered suggestive of PA. PA was confirmed with saline infusion test. Adrenal vein sampling (AVS) was conducted to identify bilateral or unilateral subtypes of PA.

#### 2.4.4. Pheochromocytoma (PHEO)

Diagnosis of pheochromocytoma was based on elevated plasma metanephrines and normetanephrines, localization of the tumor was visualized by CT, magnetic resonance imaging (MRI), ^131^I-meta-iodobenzyl guanidine scanning, and PET-CT [12]. And final confirmation was made by pathological examination (adenoma adrenal medullary).

#### 2.4.5. Cushing's Syndrome (CS)

Cushing's syndrome was diagnosed on hormonal assessments, including nonsuppressible serum cortisol after 1 mg of overnight dexamethasone and 48 h low-dose (2 mg) dexamethasone suppression tests. The diagnosis of location was based on the measurement of ACTH levels, a 48 h high-dose (8 mg) dexamethasone suppression test, and CT/MRI. A cortisol level higher than 5 ug/dl was not considered suppressed (a positive result) [13].

#### 2.4.6. Thyroid Dysfunction

Serum thyrotropin (TSH), free thyroxine (FT4), and triiodothyronine (FT3) levels were measured. The cut-off levels for TSH were <0.35 IU/dl for hyperthyroidism and >5.5 IU/dl for hypothyroidism. The cut-off levels for FT4 were <11.5 ng/dl for hypothyroidism and >22.7 ng/dl for hyperthyroidism. The cut-off levels for FT3 were <3.0 ng/dl for hypothyroidism and >9.2 ng/dl for hyperthyroidism.

#### 2.4.7. Obstructive Sleep Apnea (OSA)

All patients suspicious of OSA underwent full-night polysomnography. Diagnosis of OSA was based on 2009 clinical guideline of American Academy of Sleep Medicine [14]. OSA was diagnosed when apnea-hypopnea index (AHI) was ≥5 events/hour sleep.

#### 2.4.8. Anxiety

Patients with excessive anxiety, fear, worry, avoidance, and compulsive rituals as well as sleeplessness, vague pains, headaches, dizziness, stomach upset, or other somatic symptoms but no parenchymal disease were considered to have anxiety disorders. The Hamilton Anxiety Scale was used to assess the degree of anxiety disorders. The diagnosis was based on clinical practice guidelines [15] and the psychiatrist's decision. Moreover, BP values achieved the target (<140/90 mmHg) after antianxiety treatment for patients diagnosed as having anxiety in our study.

#### 2.4.9. Aortic Coarctation

If patients complained of both fatigued lower extremities, pulse palpation of arteria cruralis and arteria poplitea were decreased, or if the blood pressure values for the upper extremities are greater than that in the lower ones, then aortic coarctation was considered the probable cause. Then CTA was performed for definite diagnosis of aortic coarctation in these specific subjects.

#### 2.4.10. Others

Liddle's syndrome and polycystic ovary syndrome were diagnosed according to the published literature [16, 17].

### 2.5. Statistical Analysis

Analysis was performed with the Statistical Package for the Social Sciences, version 17.0. statistical software. The results were expressed for continuous variables with a normal distribution as the mean and standard deviation (SD). Qualitative variables were expressed as frequencies. The independent Student's* t*-test was used to estimate differences between two independent groups. Comparison of frequencies was performed by the Pearson *χ*^2^ test. A* P *value < 0.05 was considered significant.

## 3. Results

3987 hypertensive patients were evaluated during the 12-month study period, of whom 3003 patients (mean age: 52 years) were suggested for further evaluation of secondary causes or coexisting diseases of hypertension, including 1597 males and 1406 females. Study flow chart was shown in Figure 1 and the general characteristics of the study population were summarized in Table 1.

Table 2 showed the detection of secondary causes and coexisting diseases of hypertension, demonstrating that 1186 (39.5%) patients had secondary causes of hypertension and coexisting diseases among 3,003 hypertensive patients. OSA was the most common, accounting for 24.7% patients (742/3003) among secondary causes and coexisting diseases, followed by PA (5.8%), PA + OSA (4.9%), RPD (1.8%), and hypothyroidism (1.1%). Endocrine hypertension was accounting for 12.1% patients (362/3003), including 10.7% of patients with PA. 281 patients with PA had bilateral excessive aldosterone production and 40 patients had unilateral excessive aldosterone production among 321 patients with PA. Less common secondary causes of hypertension were CS (4 cases, 0.10%), PHEO (3 cases, 0.1%), and hyperthyroidism (3 cases, 0.1%). Other secondary causes were Liddle's syndrome (1 case), aortic coarctation (1 case), and polycystic ovary syndrome (1 case), respectively.

**Figure 2:**
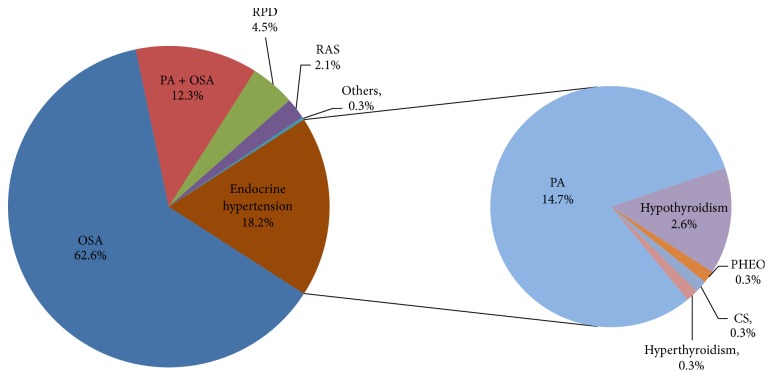
Constituent ratio in 1186 patients with secondary causes and coexisting disease. OSA, obstructive sleep apnea; PA, primary aldosteronism; RPD, renal parenchymal disease; RAS, renal artery stenosis; PHEO, pheochromocytoma; CS, Cushing's syndrome.

As given in Figure 2, OSA was present in 62.6% (742/1186) among 1186 patients of all secondary causes and coexisting diseases of hypertension and PA + OSA were present in 12.3% (146/1186) of patients. PA was the most common endocrine cause of hypertension in our population, present in 27.1% (321/1186) of patients. RPD and RAS accounted for 6.6% in total.

**Figure 3:**
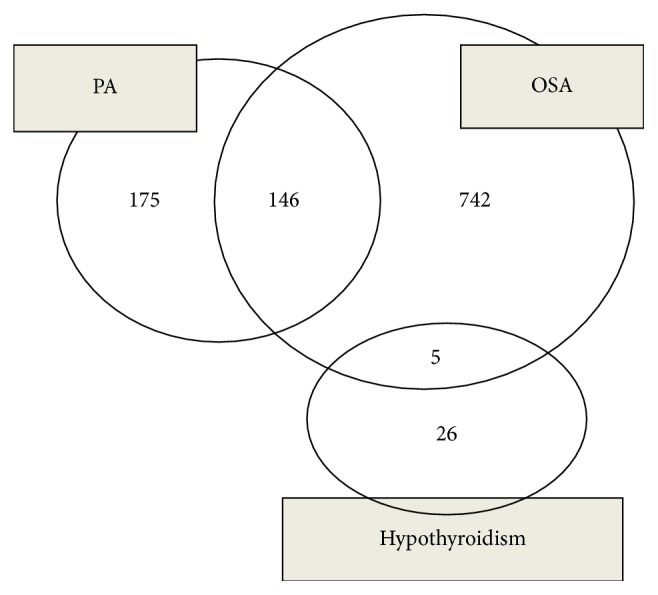
The frequency of overlapping conditions in secondary causes of hypertension. OSA, obstructive sleep apnea; RPD, renal parenchymal disease; PA, primary aldosteronism.

As shown in Figure 3, there were overlapping conditions in secondary causes and coexisting diseases of hypertension. OSA was commonly accompanied with PA; that is, 16.4% (146/888) of patients with OSA also had PA. In turn, 45.5% (146/321) of patients with PA were accompanied with various severity of OSA. Additionally, five patients with OSA had concomitant hypothyroidism.

Tables 3 and 4 presented the detection of top three secondary causes and coexisting diseases of hypertension in 3003 patients stratified via gender, age, and BMI. OSA was significantly higher in males than in females, and PA and anxiety were significantly higher in females than in males. OSA was the most common in each age- and BMI-stratified group, OSA and PA were significantly more prevalent in young and middle-aged subjects than in the older ones, and RPD was significantly higher in young subjects than in middle-aged and older ones, whereas no significant difference was observed in anxiety among different age groups. As BMI elevated, detection of OSA significantly increased (linear-to-linear *P* < 0.001 for both). Inversely, detection of anxiety significantly decreased along with BMI increasing (linear-to-linear test, *P* < 0.001). There was significantly higher detection of PA in nonobese subjects than in obese subjects.

## 4. Discussion

The present study systematically evaluated the secondary causes and coexisting diseases of hypertension in hypertensive patients extensive in sample size using strict protocols in the specialty center of hypertension dedicated to screening, diagnosis, and treatment service of secondary causes of hypertension in Northwest of China. The data demonstrated that the detection rate of secondary causes and coexisting diseases of hypertension, including endocrine diseases, RPD, RAS, and OSA, was 39.5% among 3003 hypertensive patients. OSA and PA were the most common secondary causes and coexisting diseases of hypertension among hypertensive patients. Previous studies described that prevalence of secondary causes of hypertension ranges from 4.7% to 10.5% [18–20] and that the most common secondary causes of hypertension are RPD and RAS [21]. In these previous studies, however, OSA was not included. Based on traditional secondary causes of hypertension such as endocrine hypertension, RPD, and RAS, detection of secondary hypertension was 11.1% in this current study, slightly higher than that in previous studies (4.7–10.5%). There exists a wide interval range in the previously reported prevalence (4.7–10.5%) of secondary hypertension, with the possible reasons that clinical characteristics of study subjects, systemic screening, or secondary hypertension and awareness of both clinicians and patients on secondary hypertension differed greatly.

Prevalence of secondary causes of hypertension has been encountered with increasing frequency in recent years. A recent study evaluating secondary causes of true resistant hypertension in patients aged 19 to 65 years showed that OSA was present in 71.2%, PA in 15.7%, and RAS in 5.4% [4]. Meanwhile, detection rate of secondary causes of hypertension increased gradually, from 14.7% in 2005 to 39.3% in 2008 in our center from 1999 to 2008 [22, 23], possibly attributable to the gradual improvement in screening skills of secondary hypertension. Constituent ratio of secondary causes of hypertension has also changed. Prevalence of OSA and PA is higher in the current study than in previous data of our center, possibly indicating the underestimated prevalence of secondary causes and coexisting diseases of hypertension in the past since some patients with secondary hypertension may not have been identified successfully.

OSA is considered an important independent risk factor for cardiovascular diseases in general, especially for hypertension [24], and prominent in European and American guidelines as an identifiable and treatable cause of secondary hypertension [6, 25, 26]. Evidence is gradually accumulating that OSA is common in the condition of hypertension, ranging from 37 to 56% [27, 28], and even higher among patients with resistant hypertension, reportedly between 70 and 83% [29, 30]. In a prospective cohort study, subjects with moderate to severe OSA (AHI ≥ 15/h) showed a 3.2-fold increase in the odds of developing hypertension, compared with subjects without OSA [31]. OSA is seen in 71% of those with resistant hypertension versus 38% of those with controlled hypertension [29]. Some studies also showed that treatment of OSA with continuous positive airway pressure induces significant BP reduction [32, 33]. Our data also shows that OSA is the most common secondary cause of hypertension in total subjects and in all age- and BMI-stratified groups.

Consistent with previous studies, present study also observed that PA is diagnosed in 10.7% of hypertensive patients. Prevalence of PA was initially estimated to be 1-2%, whereas it has been recently reported to be 5–16.6% in patients with hypertension [34, 35] and 17–23% in those with resistant hypertension [34]. Such increase in prevalence of PA might be partly due to the more frequent use of ARR as screening test, particularly in patients without hypokalemia.

A novel finding in our study is that several patients had multiple secondary causes, including 146 patients with PA + OSA and 5 with OSA + hypothyroidism. Previous studies have shown that severity of OSA is related to the degree of aldosterone excess and that it is greater in resistant hypertension with hyperaldosteronism [36, 37]. Therefore, we consider hypertension a cardiovascular syndrome related to many systems and believe that hypertension can be caused through multiple mechanisms.

A recent systematic review and meta-analysis suggests that there is an association between anxiety and increased risk of hypertension [38]. Individuals with anxiety have a higher risk of hypertension than those without [39, 40]. Previous studies have reported that prevalence of anxiety is 9.5% in hypertensive patients [41]. BP in patients with anxiety reached target goal (<140/90 mmHg) after antianxiety treatment in our study. Possible reasons for comparatively lower detection of anxiety in our study might be direct reference of mental disorders to the mental specialty clinic. Similarly, hypertensive patients with RPD are referred directly to a renal clinic, consequently, decreasing its frequency in our subjects.

In the current study, OSA and PA were observed to be the most common secondary causes of hypertension and coexisting disease in males; PA were in females. Patients with OSA were characterized by male gender, younger to middle-age, and higher BMI, patients with PA were characterized by female gender, younger to middle-age, and higher BMI, and patients with anxiety were by female gender and lower BMI. Patients with RPD were seen more in males and in those with BMI < 24 kg/m^2^ in our study.

The current study, however, contains some limitations. First, specialist bias might be present since all the subjects had been referred to the hypertension specialty center. Second, this is a single center study, only showing detection of secondary causes and coexisting diseases from single hypertension center in Northwest of China. Therefore, our data may not represent the prevalence of secondary causes of hypertension in general hypertensive population. Third, the current study was not involved in a detailed investigation in all hypertensive patients. Patients with positive initial screening performed additional exams, which may have underestimated the real prevalence of several secondary forms of hypertension. Fourth, we have not flowed up these patients with secondary hypertension to observe the specific treatment effects such as continuous positive airway pressure and adrenalectomy, whereas the follow-up work is planned to be performed recently within the nearest future, and we might obtain further evidence on the effects of pathogenetic cause-specific treatment in this population.

In conclusion, this study shows that the detection rate of secondary causes and coexisting diseases in hypertensive patients is 39.5% in a specialty center for hypertension in Northwest China. OSA and PA were the common secondary causes of hypertension. Findings from the current study suggest an increasing frequency of secondary forms of hypertension, highlighting the burden of OSA and PA in hypertensive patients.

## Figures and Tables

**Table 1:** Clinical and biochemical characteristics of all subjects (*n* = 3987).

Variable	Characteristics
Age (years)	52.0 ± 13.1
Male *N*, %	2121 (53.2)
Current smoker, *N* (%)	1248 (31.3)
Current drinker, *N* (%)	1100 (27.6)
BMI, (kg/m^2^)	26.9 ± 4.0
Office SBP (mmHg)	141.7 ± 21.5
Office DBP (mmHg)	91.1 ± 14.8
Heart rate (beats per min)	77.8 ± 9.3
Serum potassium (mmol/l)	3.9 ± 0.4
Fasting blood glucose (mmol/l)	5.3 ± 1.5
TC (mmol/l)	4.5 ± 1.0
TG (mmol/l)	2.0 ± 1.5
HDL-C (mmol/l)	1.2 ± 0.5
LDL-C (mmol/l)	2.5 ± 0.7
Diabetes mellitus, *N* (%)	662 (16.6)
Coronary artery disease, *N* (%)	746 (18.7)
Stroke history, *N* (%)	1132 (28.4)
Obesity, *N* (%)	1097 (27.5)
Anxiety, *N* (%)	140 (3.5)

**Table 2:** Detection of secondary causes and coexisting diseases in hypertensive patients.

Diseases	*N*	Total, %	patients with further evaluation, %
		*n* = 3987	*n* = 3003
Secondary causes and coexisting diseases	1186	29.7	39.5
OSA	742	18.6	24.7
PA	175	4.4	5.8
PA + OSA	146	3.7	4.9
RPD	54	1.3	1.8
Hypothyroidism	31	0.8	1.1
RAS	25	0.6	0.8
PHEO	3	0.08	0.1
CS	4	0.1	0.1
Hyperthyroidism	3	0.08	0.1
Others	3	0.08	0.1

PA, primary aldosteronism; OSA, obstructive sleep apnea; RPD, renal parenchymal disease; RAS, renal artery stenosis; PHEO, pheochromocytoma; CS, Cushing's syndrome; others include one Liddle's syndrome, one aortic coarctation, and one multicystic ovary syndrome.

**Table 3:** Detection of top three secondary causes of hypertension in 3003 patients stratified via gender, age, and BMI.

Group	OSA	PA	RPD
	*n* = 742	*n* = 175	*n* = 54
Gender			
Male (*n* = 1597)	556 (34.8)	85 (5.3)	37 (2.3)
Female (*n* = 1406)	186 (13.2)	90 (6.4)	17 (1.2)
*P*	<0.001	0.02	0.02
Age group			
Age < 45 (*n* = 925)	279 (30.2)	64 (6.9)	39 (4.2)
45 ≤ age < 65 (*n* = 1481)	399 (26.9)	98 (6.6)	10 (0.7)^$^
Age ≥ 65 (*n* = 597)	64 (10.7)^*∗*#^	13 (2.2)^*∗*#^	5 (0.8)^*∗*^
*P*	<0.001	<0.001	<0.001
Obese status			
BMI < 24 (*n* = 663)	57 (8.6)	49 (7.4)	20 (3.0)
24 ≤ BMI < 28 (*n* = 1243)	293 (23.6)^♀^	81 (6.5)	22 (1.8)
BMI ≥ 28 (*n* = 1097)	392 (35.7)^&*♮*^	46 (4.2)^&*♮*^	12 (1.1)^&^
*P*	<0.001	0.01	0.01

^*∗*^ *P* < 0.01: age < 45 versus age ≥ 65 group; ^&^*P* < 0.01: BMI < 24 versus BMI ≥ 28 group; ^#^*P* < 0.01: 45 ≤ age < 65 versus age ≥ 65 group; ^*♮*^*P* < 0.01: 24 ≤ BMI < 28 versus BMI ≥ 28 group; ^$^*P* < 0.01: age < 45 versus 45 ≤ age < 65 group; ^♀^*P* < 0.01: BMI < 24 versus 24 ≤ BMI < 28 group; PA, primary aldosteronism; OSA, obstructive sleep apnea; RPD, renal parenchymal disease.

**Table 4:** Detection of coexisting conditions of hypertension in 3003 patients stratified via gender, age, and BMI.

group	Anxiety	Diabetes	Obesity
	*n* = 140	*n* = 611	*n* = 1057
Gender			
Male (*n* = 1597)	35 (2.2)	334 (20.9)	626 (39.2)
Female (*n* = 1406)	105 (7.5)	277 (19.7)	431 (30.7)
*P*	<0.001	0.41	<0.001
Age group			
Age < 45 (*n* = 925)	40 (4.3)	99 (10.7)	366 (39.6)
45 ≤ age < 65 (*n* = 1481)	67 (4.5)	345 (23.3)^$^	532 (35.9)
Age ≥ 65 (*n* = 597)	33 (5.5)	167 (28.0)^*∗*^	159 (26.6)^*∗*#^
*P*	0.52	<0.001	<0.001
Obese status			
BMI < 24 (*n* = 663)	57(8.6)	102 (15.4)	—
24 ≤ BMI < 28 (*n* = 1243)	56(4.5)^♀^	236 (19.0)	—
BMI ≥ 28 (*n* = 1097)	27 (2.5)^&*♮*^	273 (24.9)^&*♮*^	—
*P*	<0.001	<0.001	

^*∗*^ *P* < 0.01: age < 45 versus age ≥ 65 group; ^&^*P* < 0.01: BMI < 24 versus BMI ≥ 28 group; ^#^*P* < 0.01: 45 ≤ age < 65 versus age ≥ 65 group; ^*♮*^*P* < 0.01: 24 ≤ BMI < 28 versus BMI ≥ 28 group; ^$^*P* < 0.01: age < 45 versus 45 ≤ age < 65 group; ^♀^*P* < 0.01: BMI < 24 versus 24 ≤ BMI < 28 group.
